# Multicomponent Conjugates of Anticancer Drugs and Monoclonal Antibody with PAMAM Dendrimers to Increase Efficacy of HER-2 Positive Breast Cancer Therapy

**DOI:** 10.1007/s11095-019-2683-7

**Published:** 2019-09-03

**Authors:** Monika Marcinkowska, Maciej Stanczyk, Anna Janaszewska, Ewelina Sobierajska, Arkadiusz Chworos, Barbara Klajnert-Maculewicz

**Affiliations:** 1 Department of General Biophysics, Faculty of Biology and Environmental Protection, University of Lodz, Pomorska 141/143, 90-236 Lodz, Poland; 2 Department of Surgical Oncology, Cancer Center, Copernicus Memorial Hospital, Lodz, Poland; 3 Centre of Molecular and Macromolecular Studies, Polish Academy of Sciences, Sienkiewicza 112, 90-236 Lodz, Poland; 4 Leibniz-Institut für Polymerforschung Dresden e.V, Hohe Strasse 6, 01069 Dresden, Germany

**Keywords:** docetaxel, HER-2, paclitaxel, PAMAM dendrimer, trastuzumab, tumour targeting

## Abstract

**Purpose:**

Conjugation of nanocarriers with antibodies that bind to specific membrane receptors that are overexpressed in cancer cells enables targeted delivery. In the present study, we developed and synthesised two PAMAM dendrimer-trastuzumab conjugates that carried docetaxel or paclitaxel, specifically targeted to cells which overexpressed HER-2.

**Methods:**

The ^1^H NMR, ^13^C NMR, FTIR and RP-HPLC were used to analyse the characteristics of the products and assess their purity. The toxicity of PAMAM-trastuzumab, PAMAM-doc-trastuzumab and PAMAM-ptx-trastuzumab conjugates was determined using MTT assay and compared with free trastuzumab, docetaxel and paclitaxel toward HER-2-positive (SKBR-3) and negative (MCF-7) human breast cancer cell lines. The cellular uptake and internal localisation were studied using flow cytometry and confocal microscopy, respectively.

**Results:**

The PAMAM-drug-trastuzumab conjugates in particular showed extremely high toxicity toward the HER-2-positive SKBR-3 cells and very low toxicity towards to HER-2-negative MCF-7 cells. As expected, the HER-2-positive SKBR-3 cell line accumulated trastuzumab from both conjugates rapidly; but surprisingly, although a large amount of PAMAM-ptx-trastuzumab conjugate was observed in the HER-2-negative MCF-7 cells. Confocal microscopy confirmed the intracellular localisation of analysed compounds. The key result of fluorescent imaging was the identification of strong selective binding of the PAMAM-doc-trastuzumab conjugate with HER-2-positive SKBR-3 cells only.

**Conclusions:**

Our results confirm the high selectivity of PAMAM-doc-trastuzumab and PAMAM-ptx-trastuzumab conjugates for HER-2-positive cells, and demonstrate the utility of trastuzumab as a targeting agent. Therefore, the analysed conjugates present an promising approach for the improvement of efficacy of targeted delivery of anticancer drugs such as docetaxel or paclitaxel.

**Electronic supplementary material:**

The online version of this article (10.1007/s11095-019-2683-7) contains supplementary material, which is available to authorized users.

## Introduction

Since their development, researchers have recognised the potential of nanocarriers as drug delivery systems. There are two strategies by which drug delivery can be achieved with nanosystems: passive delivery, which exploits the enhanced permeability and retention effect (EPR effect) to increase the penetration of nanocarriers into solid tumours; and active delivery, which is achieved by covalent conjugation of the nanocarrier to a ligand or antibody which can bind to a specific receptor that is overexpressed in cancer cells. Previous work dedicated to anticancer drug development has focused on achieving targeted delivery to the tumour, reducing adverse effects and increasing antitumour efficacy (1).

Trastuzumab is as a recombinant, humanised IG1 monoclonal antibody that selectively binds to human epidermal growth factor receptor 2 (EGFR2). Through binding to subdomain IV of the extracellular domain of overexpressed human epidermal growth factor receptor-2 (HER-2) receptors, trastuzumab blocks the receptor and inhibits excessive proliferation of HER-2-positive cancer cells. Previous research has indicated that inhibition of proliferation is a result of cell cycle arrest in the G1 phase (2). Therefore, the combination of trastuzumab and a taxane is a first-line therapy for the treatment of various cancers including lung, ovarian and metastatic breast cancer (MBC). In HER-2-positive MBC patients, three doses peer week of docetaxel (100 mg/m^2^) given in combination with trastuzumab (at a 4 mg/kg loading dose followed by 2 mg/kg once weekly) resulted in a high overall response rate (ORR), overall survival (OS), response duration, time to progression and time to treatment failure (TTF), and the toxicity of the drug combination was only slightly increased compared with docetaxel alone (3). Similarly; paclitaxel, which is the preferred single chemotherapeutic agent for recurrent or metastatic breast cancer according to National Comprehensive Cancer Network (NCCN) guidelines (4), resulted in improved progression-free survival (PFS) of HER-2-positive patients when administrated at a dosage of 80 mg/m^2^ weekly in combination with a trastuzumab monoclonal antibody at 4 mg/kg (loading dose, followed by weekly administration of 2 mg/kg), compared with paclitaxel alone (5).

The use of dendrimers as carriers of anticancer drugs or monoclonal antibodies is well known (6). Teow *et al*. demonstrated the use of a third generation (G3) polyamidoamine (PAMAM) dendrimer as a drug carrier which increased the permeability of the poorly soluble drug, paclitaxel. Cytotoxicity studies have shown that the conjugation of lauryl chains and paclitaxel to G3 dendrimers significantly (*p* < 0.05) increased the cytotoxicity of the drug toward the human Caco-2 cell line as well as primary cultures of porcine brain endothelial cells (PBECs). The conjugate showed an approximate 12-fold increase in permeability across both the apical and basolateral cell monolayers compared with paclitaxel alone (7). Paclitaxel has also been conjugated to hydroxyl-terminated PAMAM G4 dendrimers and bis-polyethylene glycol (bisPEG) polymer to achieve enhancement of drug solubility and anticancer activity. The cytotoxicity of the PAMAM dendrimer-succinic acid-paclitaxel conjugate towards A2780 human ovarian carcinoma cells was increased 10-fold compared with the free, non-conjugated drug (8). The *in vitro* studies of Miyano *et al*. have confirmed the efficacy of the monoclonal antibody conjugated to the dendrimer. The G6 PAMAM dendrimer was modified with two amino acids – lysine and glutamic acid (KG6E) – then trastuzumab and the fluorescent dye AlexaFlour 488 were attached. The results confirmed that the KG6E-trastuzumab conjugate specifically bound to SKBR-3 (HER-2-positive) cells in a dose-dependent manner, with low binding affinity for MCF-7 (HER-2-negative) cells. In addition, the conjugate was significantly internalised by SKBR-3 cells and subsequently trafficked to the lysosomes (9).

We believe that we have developed an innovative delivery system which combines both strategies. In the present study, trastuzumab was used in a PAMAM-drug-trastuzumab conjugate carrying paclitaxel (ptx) or docetaxel (doc) in order to specifically target SKBR-3 HER-2 positive cells. Over 20% of breast cancers exhibit overexpression of HER-2 human epidermal growth factor receptor-2 (HER-2) (10); therefore, targeting this receptor may represent an attractive target for nanoparticles loaded with anticancer drugs. Moreover, dendrimer conjugation significantly changes the biodistribution of low molecular weight drugs, affording the opportunity to achieve disease-specific targeting while reducing delivery to sites of toxicity (11). Our aim was to create a conjugate which will release the drugs when it is exposed to low pH, such as at the site of solid tumour, by breaking the pH-sensitive linker between the monoclonal antibody and PAMAM-drug conjugate. This will then enable passive delivery of paclitaxel or docetaxel.

For this purpose, we analysed the cytotoxicity of PAMAM-drug-trastuzumab conjugates in HER-2-positive (SKBR-3) and -negative (MCF-7) human breast cancer cells. The internalisation efficiencies and cellular trafficking were determined in order to evaluate the potential application of PAMAM dendrimers as HER-2-targeted fluorescently-labelled drug carriers. Our results indicate that PAMAM-drug-trastuzumab conjugates have increased toxicity toward HER-2-positive human breast cancer cells compared with the free drug or the PAMAM-trastuzumab conjugate. Therefore, our new conjugates could represent potential candidates for HER-2-expressing tumour targeting, and may pave the way for improvements in the effectiveness of therapy for this condition, which is the most common cancer in women.

## Materials and Methods

### Materials

Solvents for the synthesis and purification were purchased from Sigma-Aldrich. All cell culture reagents were purchased from Gibco® (Germany). Flasks and multiwell plates for *in vitro* studies were obtained from Nunc (Germany). Amine terminated PAMAM G4 dendrimer, docetaxel/paclitaxel, PBS (phosphate buffered saline), FBS (fetal bovine serum) and MTT (3-[4,5-dimethylthiazol-2-yl]-2,5-diphenyltetrazolium bromide) were purchased from Sigma-Aldrich. Trypan blue was purchased from Molecular Probes (USA). Herceptin (trastuzumab) was obtained from Roche Poland. Human breast adenocarcinoma’s cell lines: HER-2 negative (MCF-7 ATCC no. HTB-22) and HER-2 positive (SKBR-3 ATCC no. HTB-30) were purchased from ATCC (USA).

### Synthesis of PAMAM Docetaxel/Paclitaxel Conjugate

The linking of the drug to the dendrimer was done using a two steps covalent method (patent pending P.420273).

Shortly, 12.5 μmol of drug (docetaxel or paclitaxel) was dissolved in 3 ml of anhydrous DMSO at 25°C and 3-fold molar excess of *N*-(*3*-Dimethylaminopropyl)-*N′-*ethylcarbodiimide hydrochloride (EDC) was added. The mixture was stirred 0.5 h. Then 25 μmol of succinic acid was slowly added to the solution while maintaining the reaction mixing. The reaction was then stirred for 24 h. Khandare *et al*. showed that the use of EDC and other carbodiimides could be also appropriate for the synthesis of biomolecules and their functionalization with other molecules, therefore we chose the EDC/carbodiimides system instead of succinic anhydride (12). The choice of anhydrous DMSO as a synthesis solvent was determined by the high solubility of paclitaxel and docetaxel in it, as well as other reagents. Using the anhydrous DMSO, we performed the reactions in an inert gas atmosphere, as previously published (13,14).

Resulting drug-SA was stirred for 3 h at room temperature in the presence of 20 μmol of EDC. Then 1 mL of PAMAM G4 solution in methanol was evaporated in order to remove methanol and then was added to the reaction mixture and stirred at room temperature for 2 days.

The PAMAM G4-drug was purified by ultrafiltration on an Amicon Ultra-3 K (molecular weight cut-off, MWCO = 3 kDa). ^1^H NMR, ^13^C NMR and FTIR was used to analyze the purity of products and to ascertain the level of PAMAM and docetaxel/paclitaxel conjugation. ^1^H NMR and ^13^C NMR spectra were recorded on Bruker Avance III DRX-600 and 500 MHz spectrometers, using deuterated DMSO-*d6* as solvents. The FTIR spectra were collected with a FTIR ATI Mattson Spectrometer Spectrum and samples were measured as thin film in KBr crystals. The analytical data can be found in the supplementary material.

### Synthesis of PAMAM-Doc-Trastuzumab and PAMAM-ptx-Trastuzumab Conjugate

The synthesis of PAMAM-doc-trastuzumab and PAMAM-ptx-trastuzumab conjugate was performed according to the patented method (patent pending P.421440 P.420274)*.*

#### Activation of Trastuzumab

SMCC was dissolved in a small volume of DMF, and diluted by adding 0.1 M PBS (phosphate buffered saline) pH 7.6, which contains 5 mM EDTA to obtain 1 mg/ml. The solution was added to trastuzumab. The mixture was incubated for 1 h at room temperature (RT). In the next step product was purified and buffer-exchanged into PBS pH 7.0, with Amicon Ultra-30 K column (MWCO = 30 kDa).

#### Introduction of Thiol Groups for the PAMAM G4 Dendrimer Surface

Traut’s reagent converts primary amine into thiol in the range of pH 7–10, however its half-life in solution decreases as the pH increases. Modification with Traut’s reagent (*2-*iminothiolane) is very efficient and occurs rapidly at slightly basic pH. To introduce thiol groups into G4 dendrimer surface, the primary amine groups were reacted with a 10:1 mol excess of Traut’s reagent in 0.1 M PBS buffer, at room temperature under N_2_ for 1 h pH 8.0. Thiolated PAMAM G4 was purified and buffer exchanged into PBS, pH 7.0 by ultrafiltration on an Amicon Ultra-3 K column.

#### The Reaction of the Modified PAMAM G4 Dendrimer with the Activated Trastuzumab

Derivatized trastuzumab was reacted with thiolated PAMAM G4 dendrimer at a 1:12 M ratio. The reaction was conducted in PBS, pH 7.0 at 25°C for 24 h. Finally, the PAMAM-trastuzumab conjugate was purified from the excess of thiolated PAMAM G4 by Amicon Ultra-30 K (MWCO 30 kDa). The final stoichiometric ratio for PAMAM-drug-trastuzumab conjugate was 1:1:1.

Reverse phase high performance liquid chromatography (RP-HPLC) was used to analyze the purity of products and to ascertain the level of PAMAM and trastuzumab conjugation. Solvents used for HPLC analysis were at the HPLC grade; ^i^PrOH, MeOH, MeCN was from Sigma-Aldrich, trifluoroacetic acid from J.T.Baker (9470) and Milli-Q water. All experiments were performed on two FPLC/HPLC systems: (1) AKTA Purifier two pumps system equipped with UV-900 monitoring, pH and conductivity probe and fraction collector Frac-920. Analysis using AKTA was performed at room temperature 25°C, (2) Shimadzu Prominence UFLC system equipped with LC-20 AD isocratic pumps with RF-20A fluorescence detector, SPD-M20A diode array detector for UV-Vis monitoring and CTO-20ASvp column oven that was setup at 75°C. Initially SOURCE uRPC C2/C18 ST 4.6/100 column was used, but it appeared to be too hydrophobic for dendrimer and antibody analysis, therefore for all presented results Jupiter 4u Proteo 90A 2.0/100 column was used.

### Synthesis of FITC Labeled Docetaxel/Paclitaxel and PAMAM-ptx/PAMAM-Doc Conjugate

The linking of FITC to the dendrimer was done using a two steps covalent method. Shortly, 6 μmol of drug (docetaxel or paclitaxel) was dissolved in 2 ml of anhydrous DMSO at 25°C and 3-fold molar excess of EDC was added. The mixture was stirred 3 h. Then 7 μmol of FITC was slowly added to the drug solution while maintaining the reaction mixing. The reaction mixture was stirred for 24 h. To obtain PAMAM-drug-trastuzumab conjugate 5,6 μmol of PAMAM G4 was added to resulting products: FITC labeled docetaxel and FITC labeled paclitaxel and stirred at room temperature for 2 days. Then the trastuzumab was reacted and the product was purified as described earlier. The final stoichiometric ratio for drug:FITC conjugate was 1:1 and for PAMAM-drug-trastuzumab-FITC conjugate 1:1:1:1.

The conjugates contained FITC were purified by ultrafiltration on an Amicon Ultra-3 K (molecular weight cut-off, MWCO = 3 kDa). ^1^H NMR and ^13^C NMR were used to analyze the purity of products and to ascertain the level of FITC conjugation. ^1^H NMR and ^13^C NMR spectra were recorded on Bruker Avance III DRX-600 and 500 MHz spectrometers, using deuterated DMSO-*d6* as solvents.

### Cell Culture

HER-2 negative human breast adenocarcinoma (MCF-7) cell line was grown in DMEM medium supplemented with GlutaMAX and 10% (*v*/v) fetal bovine serum (FBS). HER-2 positive human breast adenocarcinoma (SKBR-3) cell line was grown in McCoy’s5 medium supplemented GlutaMAX and 10% (v/v) fetal bovine serum (FBS). Cells were cultured in T-75 culture flasks in a humidified atmosphere containing 5.0% CO_2_ at 37°C and subcultured every 2 or 3 days. Cells were harvested and used in experiments after obtaining 80–90% confluence. The number of viable cells was determined by the trypan blue exclusion assay with the use of Countess Automated Cell Counter (Invitrogen). Cells were seeded in flat bottom 96-well plates at a density of 2.0 × 10^4^ cells/well in 100 μL of an appropriate medium. After seeding, plates were incubated for 24 h in a humidified atmosphere containing 5.0% CO_2_ at 37°C in order to allow cells attaching to the plates.

### Determination of Cytotoxicity

The influence of the PAMAM dendrimer conjugates and free docetaxel or paclitaxel on the cell viability was determined with the use of the MTT-assay. Briefly, to the 96-well plates containing MCF-7 and SKBR-3 cells at the density of 2.0 × 10^4^ cells/well in appropriate medium different concentrations of all compounds were added. Cells were incubated with the dendrimer for 24 h in a 37°C humidified atmosphere containing 5.0% CO_2_. After the incubation cells were washed with phosphate buffered saline (PBS). Next, 50 μL of a 0.5 mg/mL solution of MTT in PBS was added to each well and cells were further incubated under normal culture conditions for 4 h. After incubation the residue MTT solution was removed and the obtained formazan precipitate was dissolved in DMSO (100 μL/well). The conversion of the tetrazolium salt (MTT) to a colored formazan by mitochondrial and cytosolic dehydrogenases is a marker of cell viability. Before the absorbance measurement plates were shaken for 1 min and the absorbance at 570 nm was measured using the PowerWave HT Microplate Spectrophotometer (BioTek,USA).

### Determination of Hemolysis

The influence of the PAMAM dendrimer conjugates on the hemolysis was determined with the spectrophotometric method used previously (15). Briefly, human blood from healthy adult donors was obtained from a local blood bank. Blood was centrifuged for 10 min at 400 g to remove serum and buffy coat. Next, erythrocytes were washed four times with ten volumes of PBS buffer (pH = 7.4), followed by centrifugation for 10 min at 400 g. Erythrocytes were suspended in PBS buffer, the hematocrit was measured and erythrocytes suspension was diluted to the hematocrit of 2%. Erythrocyte suspension was mixed with analyzed compounds solutions in the same buffer to obtain final 1 μM concentration. Samples were incubated for 24 h and 48 h in 37°C. Next, samples were centrifuged at 400 g for 10 min. Supernatant was removed and the absorbance of supernatant at 540 nm was measured. For positive and negative control erythrocytes suspensions in distilled water and in PBS were used, respectively. The hemolysis amount was calculated from the equation:

$$ \mathrm{Hemolysis}\%=\left({\mathrm{OD}}_{\mathrm{sample}}-{\mathrm{OD}}_{\mathrm{negative}\ \mathrm{control}}\right)/\left({\mathrm{OD}}_{\mathrm{positive}\ \mathrm{control}}-{\mathrm{OD}}_{\mathrm{negative}\ \mathrm{control}}\right)\times 100\% $$

For comparison, the same experiment was performed for free docetaxel and paclitaxel.

### Cellular Uptake Detection

*In vitro* uptake studies were carried out using FITC labeled docetaxel or paclitaxel and PAMAM-doc-trastuzumab or PAMAM-ptx-trastuzumab conjugate. Compounds were added at a final concentration of 0.1 μM to the 12-well plates containing MCF-7 and SKBR-3 cells at the density of 1.5 × 10^4^ cells/well. In this study cells were incubated with the compounds for a specific time in a range from 1 h to 48 h in humidified atmosphere containing 5.0% CO_2_ at 37°C. After the appropriate incubation cells were washed with PBS, suspended in 500 μL of medium and immediately analyzed with a Becton Dickinson LSR II flow cytometer (BD Biosciences, USA) using a blue laser - 488 nm and PE bandpass filter – 575/26 nm.

### Confocal Microscopy

Confocal microscopy images were obtained with confocal inverted microscope SP-8, Leica equipped with 405 nm laser (Leica, DE). Cells at the density of 1 × 10^4^ cells/well (SKBR-3) and 0.75 × 10^4^ cells/well (MCF-7) were seeded on 96-well glass-bottom plates and incubated with 0.1 μM FITC labeled docetaxel or paclitaxel or PAMAM-doc-trastuzumab or PAMAM-ptx-trastuzumab conjugate for 24 h in 37°C humidified atmosphere containing 5.0% CO_2_. After the incubation, cells were cooled on ice and washed once with cold phosphate buffered saline (PBS) to inhibit endocytosis. Cells were imaged to visualize fluorescence of FITC labeled docetaxel or paclitaxel in green channel (excitation 488 nm, emission 520 nm) and in transmitted light.

### Statistical Analysis

Data was expressed as mean ± SD. Analysis of variance (ANOVA) with the Tukey post hoc test was used for results comparison. All statistics were calculated using the Statistica software (StatSoft, Tulsa, USA), and *p* values <0.05 were considered significant.

## Results and Discussion

### Synthesis and Characterisation of the Conjugates

**Fig. 1:**
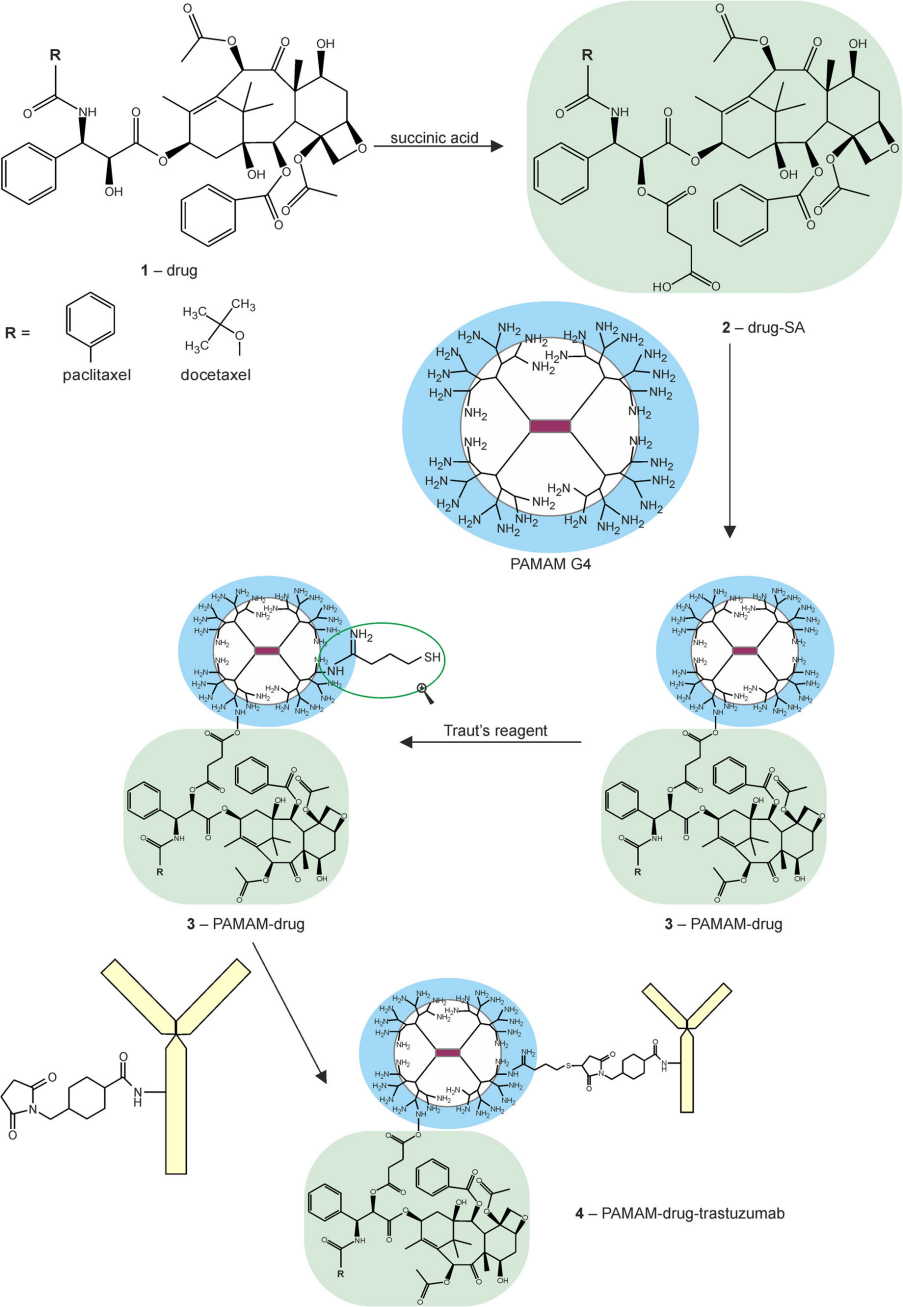
Synthesis of the PAMAM-drug-trastuzumab conjugate

We have developed an innovative delivery system consisting of three components, each of which plays a different role. Trastuzumab provides specificity against human epidermal growth factor receptor 2 (HER-2), which is overexpressesed in various cancers including breast cancer; taxanes (docetaxel and paclitaxel) provide cytotoxic effects and the PAMAM dendrimer protects the whole conjugate in the circulatory system and provides specific drug release in the tumour environment when linked with an anticancer drug via a pH-sensitive linker. Yabbarov *et al*. has confirmed the dependence drug release on decreasing pH (16). He observed that pH-dependent linkages are hydrolysed in the environment of the tumour to release the drug, which enables controlled administration of the active substance at the chosen site by exploiting the natural properties of tumour cells: high metabolism and acidic pH. We decided to link docetaxel or paclitaxel to the PAMAM dendrimer using succinic acid (SA) (17). Figure 1 illustrates the steps of synthesis of the PAMAM-drug-trastuzumab conjugate.

The chemical structures of PAMAM-doc and PAMAM-ptx were characterised by ^1^H NMR, ^13^C NMR analysis and FTIR spectroscopy (analytical data can be found in the supplementary material).

**Fig. 2:**
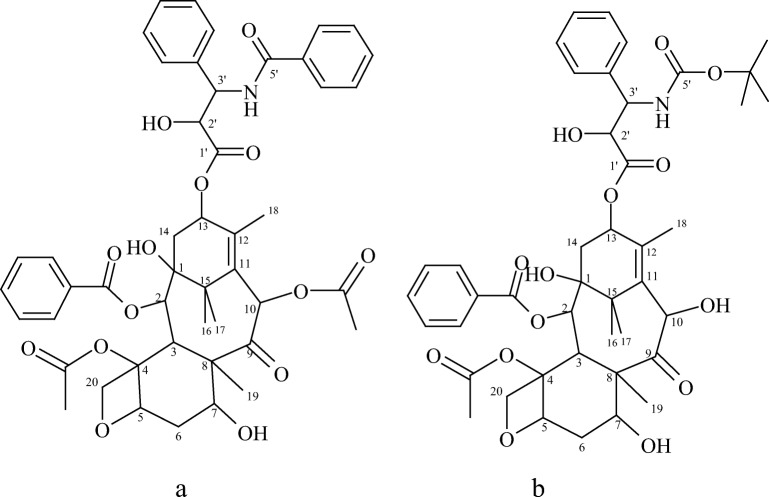
The structures of drugs: (**a**) paclitaxel, (**b**) docetaxel

For clarity of ^1^H-NMR and ^13^C-NMR spectra, Fig. 2 presents the structures of drugs (a. paclitaxel, b. docetaxel) with the marked number of carbon atoms.

The structure of paclitaxel-FITC, PAMAM-ptx-FITC, docetaxel-FITC and PAMAM-doc-FITC was confirmed by their ^1^H-NMR spectra using 500 MHz Bruker AVANCE instrument in 310 K. Chemical shifts are reported in ppm downfield from TMS using DMSO-*d6* as a solvent.

**Fig. 3:**
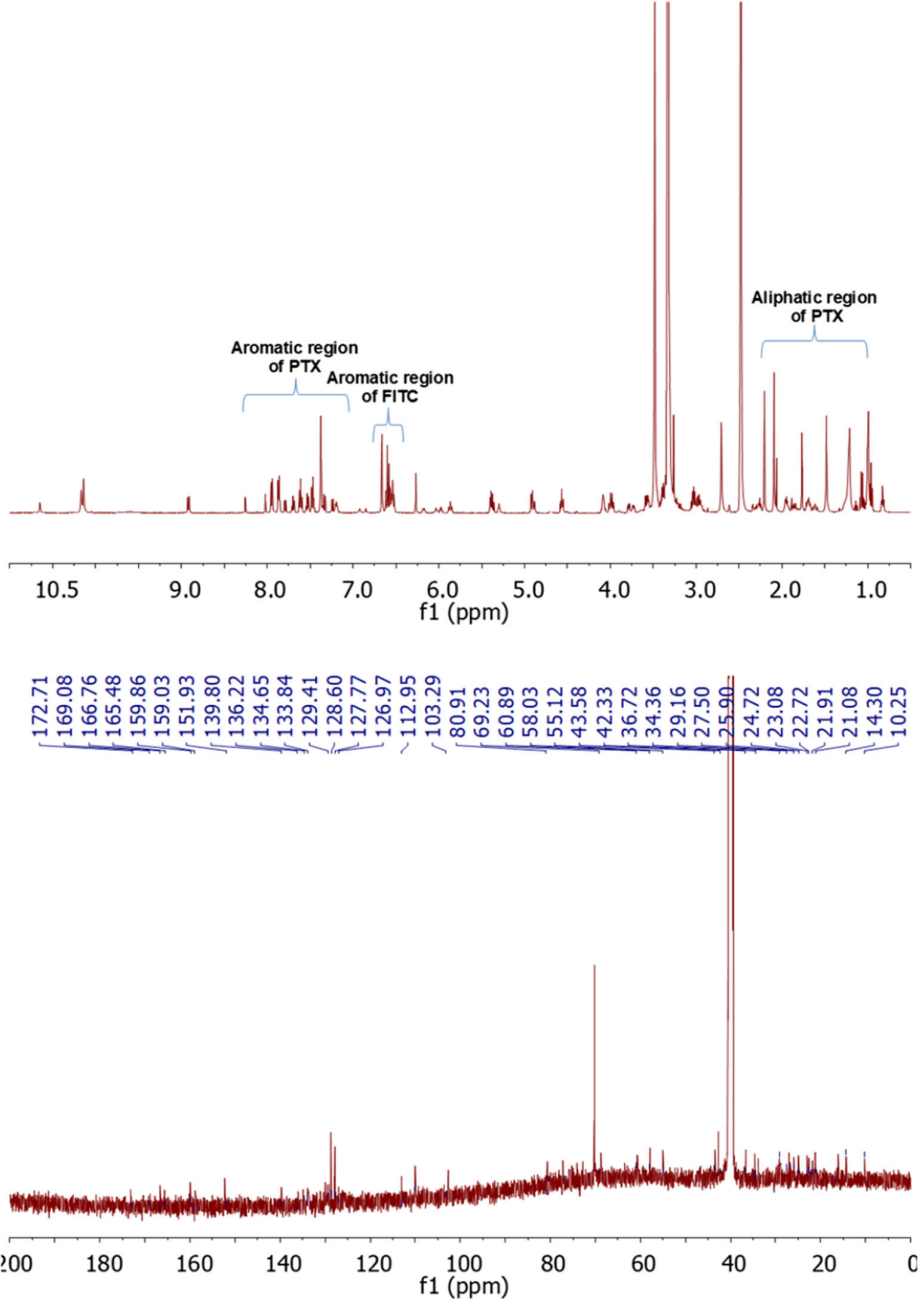
The ^1^H-NMR spectrum of paclitaxel-FITC (upper panel) and the ^13^C-NMR spectrum of paclitaxel-FITC (lower panel)

Figure 3 (upper panel) presents the ^1^H-NMR spectrum for paclitaxel-FITC. Proton signals occurring for drug appear for H_17_ at 0.99 ppm, H_16_ at 1.09 ppm, H_19_ at 1.48 ppm, H_18_ at 1.77 ppm, H_14_ at 1.89 ppm, H_10_ at 2.09 ppm, H_4_ at 2.21 ppm, H_3_ at 3.58 ppm, H_20_ at 4.01 ppm, H_7_ at 4.09 ppm, OH_1_ at 4.69 ppm, H_5_ at 4.90 ppm, H_3’_ at 5.37 ppm, H_2_ at 5.40 ppm, H_2’_ at 4.55 ppm, H_13_ at 5.88 ppm, H_10_ at 6.27 ppm, m-Bz_2_ at 7.61 ppm, p-Bz_2_ at 7.71 ppm, o-Bz_2_ at 7.96 ppm, o-NHBz at 7.48 ppm, o-NHBz at 7.55 ppm, o-NHBz at 7.83 ppm, p-Ph_3’_ at 7.23 ppm, o-Ph_3_’ at 7.37 ppm, m-Ph_3’_ at 7.37 ppm, NH at 8.93 ppm. Aromatic signals for FITC (associated with aromatic protons atom adjacent to phenol group) appear at 6.52–6.62 ppm. Signals from –NHCS- appear at 10.65 ppm. Moreover, H_2’_ proton peak on the ^1^H NMR spectra was shifted to 5.87 ppm. In the ^13^CNMR spectrum (Fig. 3 lower panel) we observed chemical shift of C2’ to 80.91 ppm.

**Fig. 4:**
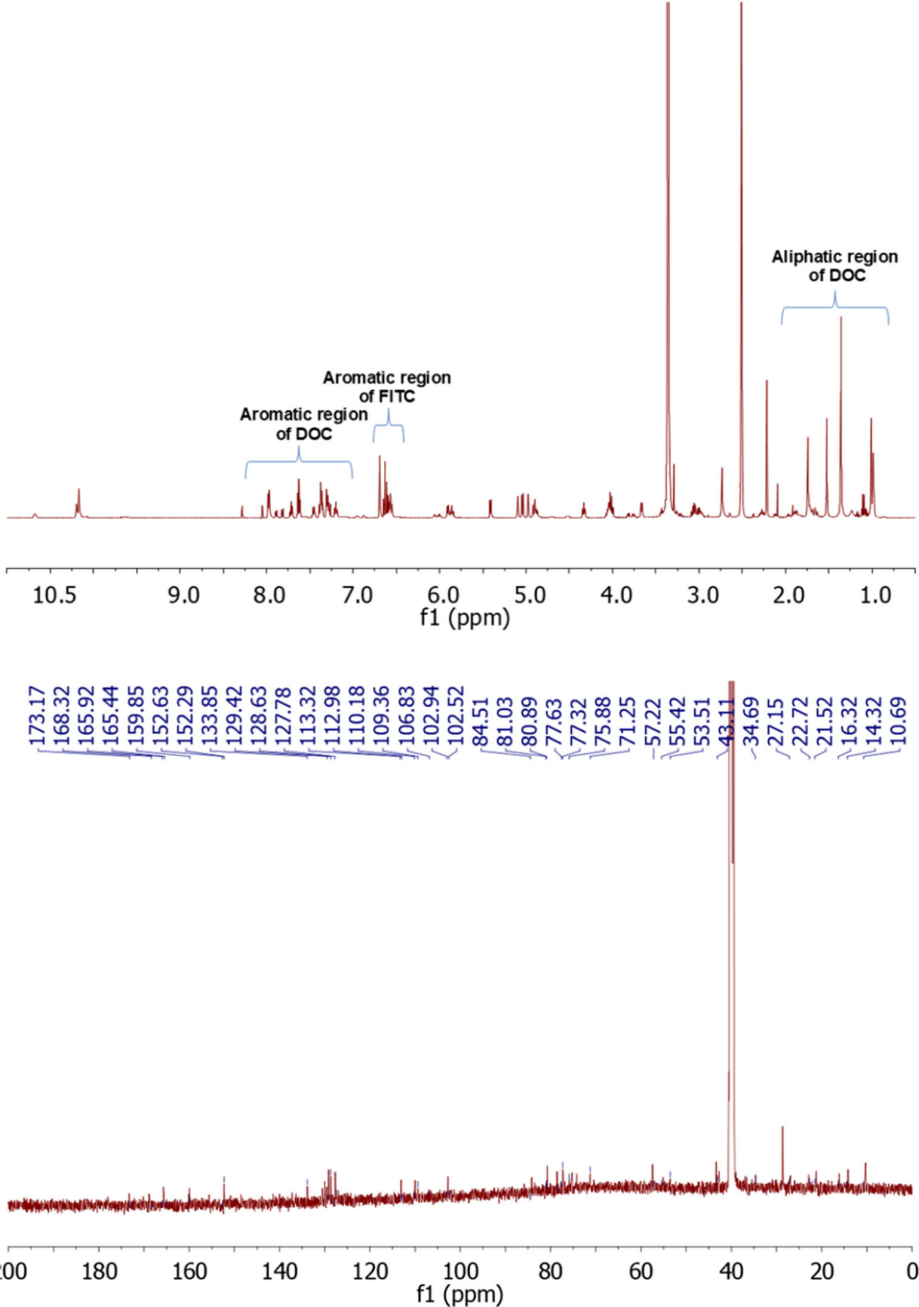
The ^1^H NMR spectrum of docetaxel-FITC (upper panel) and the ^13^C-NMR spectrum of docetaxel-FITC (lower panel)

Figure 4 (upper panel) presents the ^1^H-NMR spectrum for docetaxel-FITC. Proton signals occurring for drug appear for H_17_ at 0.99 ppm, H_16_ at 1.01 ppm, OC-CH3 at 1.36 ppm, H_19_ at 1.52 ppm, H_18_ and H_14_ at 1.74 ppm, H_6_ at 1.92 ppm, -O-CO-CH_3_ at 2.28 ppm, H_3_ at 3.66 ppm, H_20_ at 4.03 ppm, H_7_ at 4.33 ppm, H_5_ at 4.90 ppm, H_10_ at 4.98 ppm, H_3’_ at 5.04 ppm, NH at 5.09 ppm, H_2_ at 5.42 ppm, C_13_ at 5.90 ppm, o-, m-, p- Ph_3’_ at 7.37 ppm, m-Bz_2_ at 7.62 ppm, p-Bz_2_ at 7.71 ppm, o-Bz_2_ at 7.97 ppm. Aromatic signals for FITC (associated with aromatic protons atom adjacent to phenol group) appear at 6.55–6.70 ppm and 7.81–790 ppm. Signals from –NHCS- appear at 10.68 ppm. Moreover, H_2’_ proton peak on the ^1^H NMR spectra was shifted to 5.86 ppm. In the ^13^CNMR spectrum (Fig. 4 lower panel) we observed chemical shift of C2’ to 80.89 ppm.

**Fig. 5:**
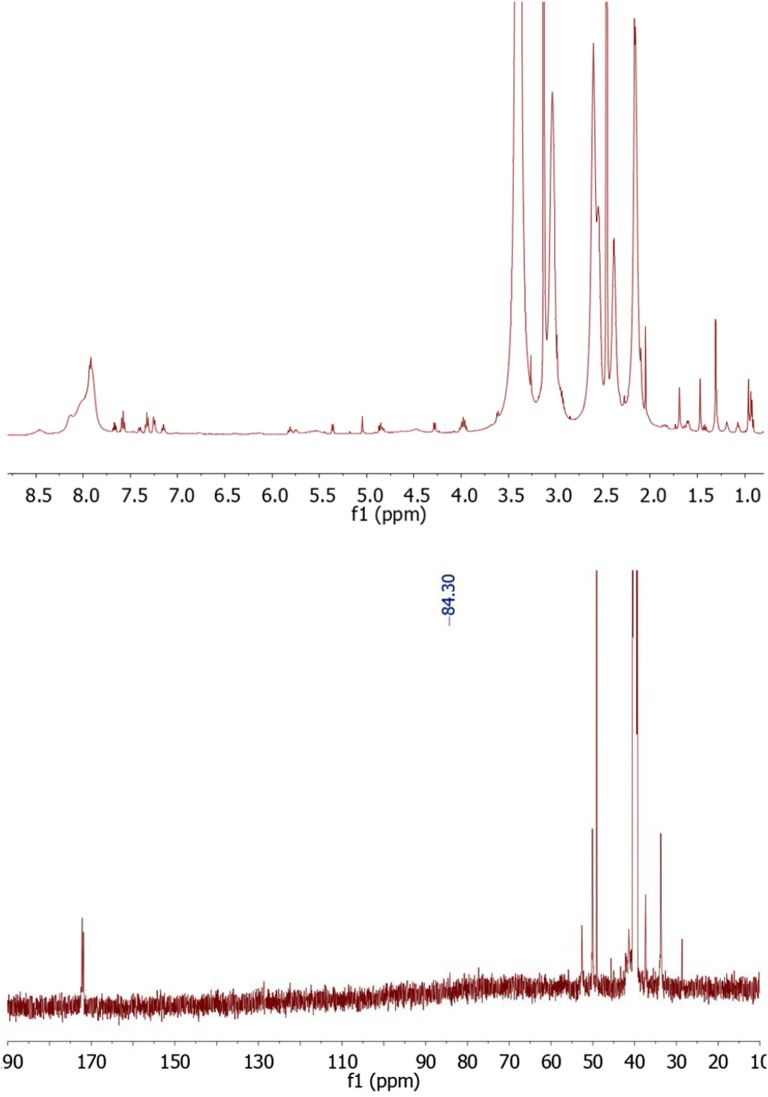
The ^1^H NMR spectrum of PAMAM-ptx-FITC (upper panel) and the ^13^C-NMR spectrum of PAMAM-ptx-FITC (lower panel)

Figure 5 (upper panel) presents the ^1^H-NMR spectrum for PAMAM-ptx-FITC (DMSO-d6, 300 MHz, ppm). Proton signals for PAMAM dendrimer appear at 2.17 ppm for –C**H**_**2**_–C(O)–NH, 2.38 ppm for –C**H**_**2**_–N–, 2.55–2-60 ppm for –N–C**H**_**2**_–, 3.04–3.13 ppm for –C**H**_**2**_–NH_2_ and –C(O)NH–C**H**_**2**_, 7.92 ppm for –CON**H**. Aromatic signals for paclitaxel and FITC appear at (δ (ppm) ≈ 7.14–7.65). Moreover, H_2’_ proton peak for paclitaxel was shifted to 5.81 ppm, and signal for NH group at 8.48 ppm. The number of paclitaxel molecules conjugated with PAMAM dendrimer was calculated using the proton integration method. Unfortunately, it is very difficult to confirm the results of conjugation by ^13^C NMR spectrum (Fig. 5 lower panel) due the small concentration of the sample and too big molar mass difference between the drug and the PAMAM dendrimer. We can only presume that signal at 84.30 comes from the bond between the drug and the dendrimer.

**Fig. 6:**
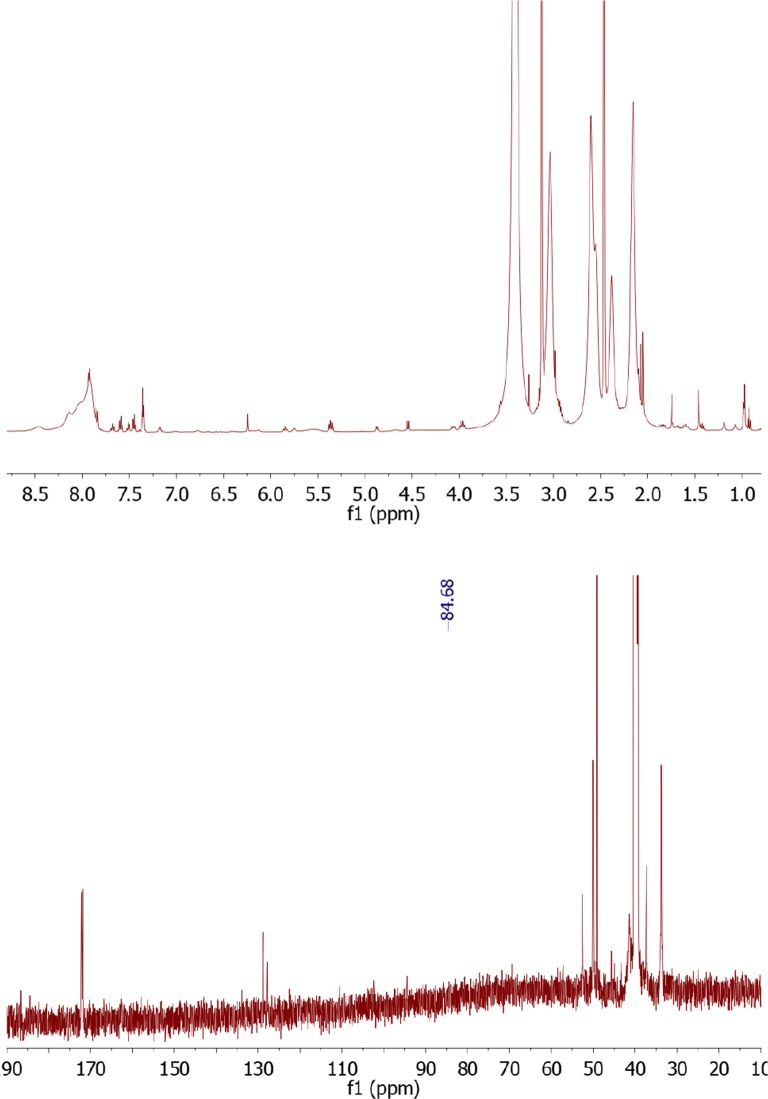
The ^1^H NMR spectrum of PAMAM-doc-FITC (upper panel) and the ^13^C-NMR spectrum of PAMAM-doc-FITC (lower panel)

Figure 6 (upper panel) presents the ^1^H-NMR spectrum for PAMAM-doc-FITC (DMSO-d6, 300 MHz, ppm). Proton signals for PAMAM dendrimer appear at 2.16 ppm for –CH2–C(O)–NH, 2.38 ppm for –CH2–N–, 2.55–2-60 ppm for –N–CH2–, 3.04–3.13 ppm for –CH2–NH2 and –C(O)NH–CH2, 7.92 ppm for –CONH. Aromatic signals for docetaxel and FITC appear at (δ (ppm) ≈ 7.17–7.69). Moreover, H2’ proton peak of docetaxel was shifted to 5.84 ppm, and signal for NH group at 8.46 ppm. The number of docetaxel molecules that conjugated with PAMAM dendrimer was calculated using the proton integration method. Also in this case ^13^C NMR spectrum (Fig. 6 lower panel), due the small concentration of sample and too big molar mass difference between the drug and the PAMAM dendrimer, allows only to presume that the signal at 84.68 comes from the bond between docetaxel and the PAMAM dendrimer.

In the next step, PAMAM-doc and PAMAM-ptx conjugates were conjugated to the monoclonal antibody (trastuzumab). To accomplish this, we used a succinimidyl 4-(N*-*maleimidomethyl)cyclohexane-1*-*carboxylate (SMCC) linker, which provides a convenient crosslinking agent for amino and thiol groups. The NHS esters react with primary amines to form stable amide bonds and the maleimide part reacts with sulfhydryl groups to form stable thioethers. To carry out the crosslinking reaction, we modified the amine groups of the PAMAM dendrimer into thiols using *2-*iminothiolane (Traut’s reagent). Modification with Traut’s reagent is very efficient and rapid at a slightly basic pH (18).

**Fig. 7:**
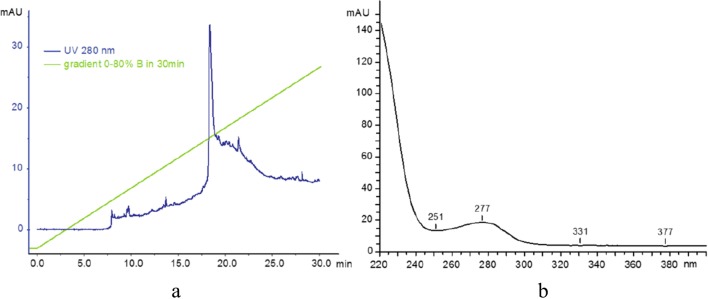
(**a**) Reverse-phase high performance liquid chromatography profile of trastuzumab. (**b**) Ultraviolet profile of the main signal at 18 min

The conjugates were characterised using FPLC/HPLC analysis. Reverse-phase high performance liquid chromatography (RP-HPLC) was used to analyse the purity of products and to ascertain the degree of PAMAM and trastuzumab conjugation. Initially, a water/acetonitrile elution system was used, but improved performance was achieved with the modified buffer: A: 0.1% TFA in water, B: 70% ^i^PrOH, 20% MeCN, 0.1% TFA in water. Elution was typically performed using a gradient of 0–80% B over 30 min, followed by 80–100% B in 5 min, then 100% B for 10 min and finally 100–0% B in 5 min (Fig. 7a). Samples were typically injected as 20–100 μg of material suspended in 100 μL of buffer A. The PAMAM dendrimer has been reported to absorb at 214 nm, and so absorbance at this wavelength along with 280 nm (to detect protein) were recorded. We also monitored absorbance at 254 nm to measure any potential contamination. Additionally, a Shimadzu diode array system provided UV profiles (recorded at 200–600 nm). This allowed the purity of PAMAM dendrimer to be ascertained, by its absorbance at 220 nm has also been reported previously. The analytical data can be found in the supplementary material.

In the second step, analysis of trastuzumab was carried out. Analysis was performed on a UFLC system (composed of two LC-20ADXP isocratic pumps, a CTO-20AS column oven with diode array UV-Vis monitoring) operated at 75°C. The elution system was as before (A: 0.1% TFA in water, B: 70% ^i^PrOH, 20% MeCN, 0.1% TFA in water). The system was run at a gradient of 0–80% B over 30 min to elute the main product (monitored at 280 nm), which appeared at 18.3 min. The UV profile of the main signal showed absorbance at 277 nm, as is expected for proteins (Fig. 7b).

**Fig. 8:**
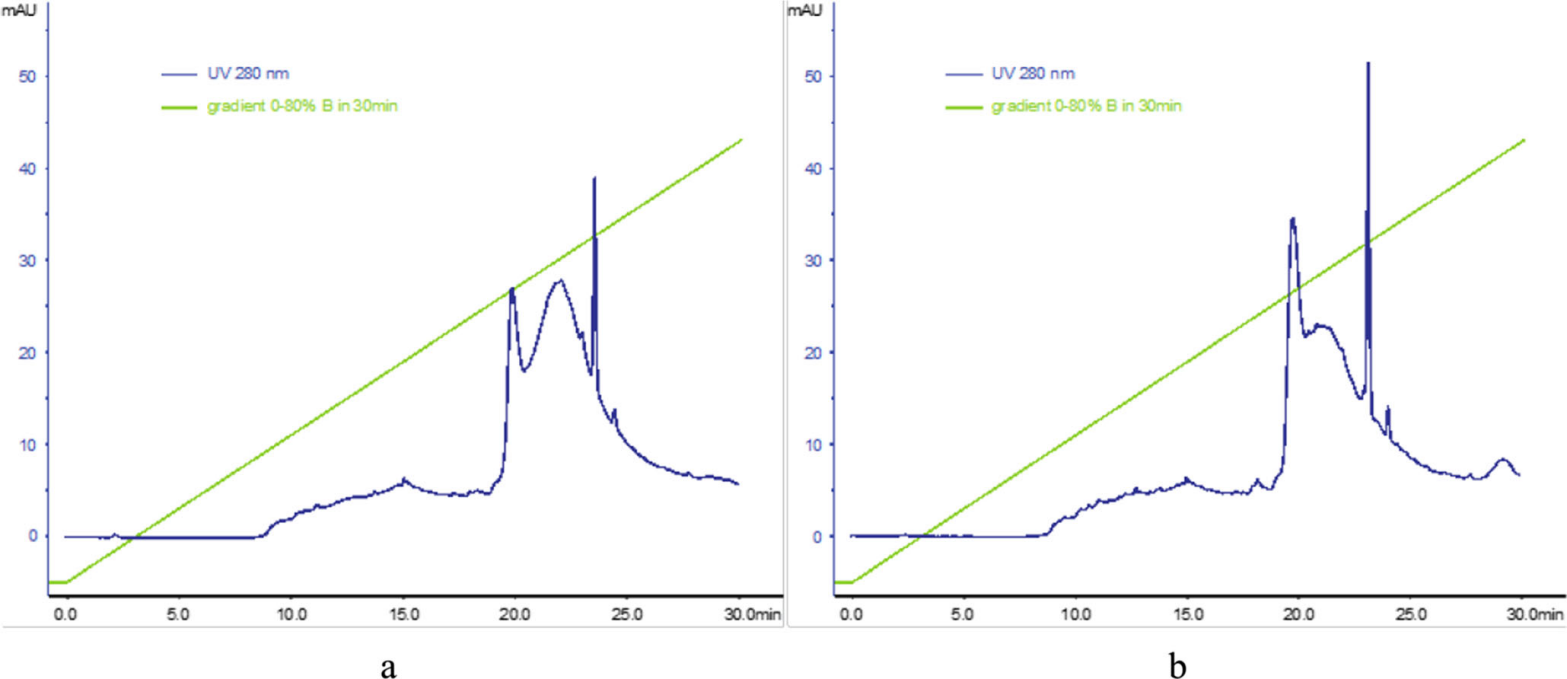
Reverse-phase high performance liquid chromatography profiles of (A) PAMAM-doc-trastuzumab conjugate (**a**) and PAMAM-ptx-trastuzumab conjugate (**b**)

Finally, the PAMAM-doc-trastuzumab and PAMAM-ptx-trastuzumab conjugates were analysed. Analysis of the chromatography profiles showed absorption at 280 nm. Analysis was carried out as before at 75°C, using buffers A: 0.1% TFA in water, B: 70% ^i^PrOH, 20% MeCN, 0.1% TFA in water; and a gradient of 0–80% B over 30 min, following injection of 100 μg of sample. The UV profile of the signal at 21 min shows three peaks (Fig. 8). As expected, absorbance signals were observed at 276, 504 and 540 nm, which are characteristic of proteins, docetaxel or paclitaxel, respectively.

### *In Vitro* Studies

**Fig. 9:**
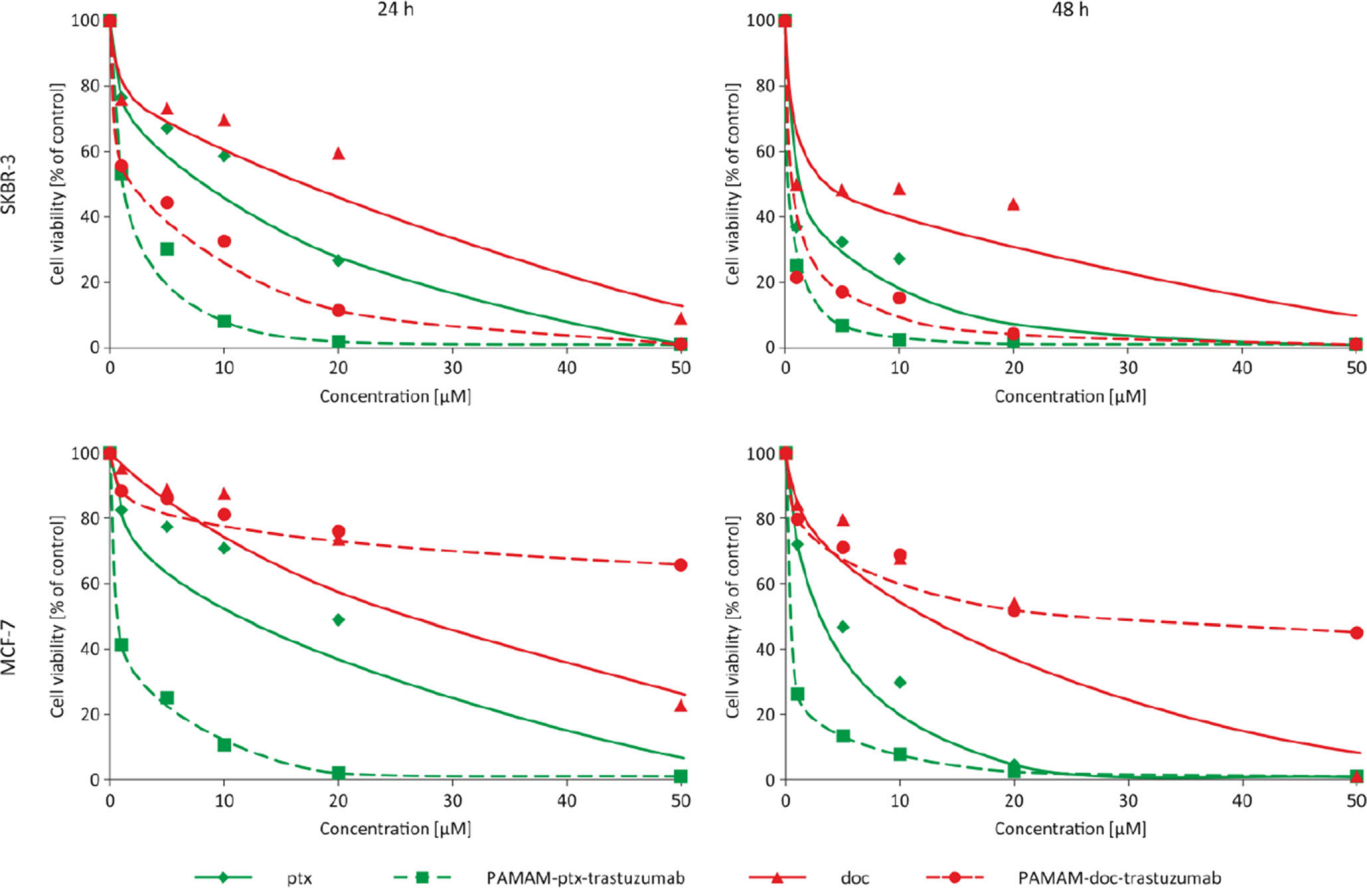
Influence of paclitaxel (green rhombus), docetaxel (red triangles), PAMAM-ptx-trastuzumab (green squares) and PAMAM-doc-trastuzumab (red spheres) conjugates on the viability of MCF-7 and SKBR-3 cells as assessed by MTT assay

There are examples of PAMAM dendrimer conjugated with various anticancer drugs reported (16,17), but few involve the use of a monoclonal antibody as a disease-specific targeting agent (18–20). In this study, the PAMAM dendrimer was conjugated to trastuzumab using a pH-dependent linker which can be applied for drug conjugation because the linker-bonded conjugates are stable in the extracellular media. This ensures low drug release, but lability when the conjugates enter the lysosomes which allows release of the drug to elicit its antitumour activity (11,21). In the present study, the number of drug molecules per PAMAM dendrimer molecule was calculated to be 1.0. The biocompatibility of the PAMAM-doc-trastuzumab and PAMAM-ptx-trastuzumab conjugates was evaluated using the MTT assay to measure the cytotoxicity in two different breast cancer cell lines: HER-2-negative human breast adenocarcinoma (MCF-7) and HER-2-positive human breast adenocarcinoma (SKBR-3). Measurements were made after 24 and 48 h of incubation and after a 24-h incubation with the drug, removal of drug, and another 24-h incubation without drug (24-24 h). The addition of this incubation variant allows to assess cell damage and mortality after the drug removing from the system. Figure 9 shows the cell viability profiles that were obtained for each conjugate compared with the free drug for both cell lines. The cytotoxicity of docetaxel and paclitaxel was dose-dependent, with IC_50_ values of around 23.7 and 7.8 μM (respectively) after a 24-h incubation in the MCF-7 cell line, and 10.7 and 7.3 μM (respectively) after a 24-h incubation in the SKBR-3 cell line. In contrast, trastuzumab itself exhibited very low toxicity even toward SKBR-3 cells, with cell viability observed to be over 85% following exposure to 20 μM concentration of the drug. However, conjugation of antibody with PAMAM dendrimer improved that cytotoxic effect (results published earlier (14)). The observed effect was more evident for SKBR-3 cells than MCF-7 cells, due to selective binding of the conjugate to cells that overexpress HER-2. These results are in good agreement with previous studies (Miyano *et al*.), that showed that when the glutamate-modified sixth generation lysine dendrimer (KG6E) was conjugated with trastuzumab, binding with the HER-2 receptor was more specific and exhibited a higher cellular internalisation rate compared with the free monoclonal antibody (9). Other studies have confirmed the lack of antiproliferative activity of free trastuzumab toward different HER-2-positive cell lines (22).

Importantly, the addition of taxanes to the PAMAM-trastuzumab conjugate enhanced the therapeutic effect and selectivity of the conjugates in comparison with the free drugs. This was particularly obvious after 48 h of incubation and after 24-24 h. The IC_50_ values (Table I) in SKBR-3 cells indicate that both PAMAM-drug-trastuzumab conjugates showed increased selectivity and therapeutic effects compared with the free drugs, but the most remarkable result of the present study is the selectivity that was observed between cell lines. The PAMAM-doc-trastuzumab conjugate in particular showed extremely high toxicity toward the HER-2-positive SKBR-3 cells and very low toxicity towards to HER-2-negative MCF-7 cells.

**Table I:** Comparison of IC_50_ values for paclitaxel, docetaxel, PAMAM-ptx-trastuzumab and PAMAM-doc-trastuzumab conjugates in two breast cancer cell lines

	MCF-7 24 h	MCF-7 24 h–24 h	MCF-7 48 h	SKBR-3 24 h	SKBR-3 24 h–24 h	SKBR-3 48 h
Ptx	7.82 ± 0.18	3.88 ± 0.74	2.24 ± 0.33	7.31 ± 1.54	0.30 ± 0.03†	0.49 ± 0.13†
PAMAM-ptx-trastuzumab	0.585 ± 0.18*	0.05 ± 0.01*	0.09 ± 0.01*	0.72 ± 0.21*	0.005 ± 0.004*†	0.002 ± 0.001*†
Doc	23.76 ± 4.81	21.47 ± 14.42	9.19 ± 3.36	10.75 ± 1.50†	2.85 ± 0.04	2.00 ± 0.44
PAMAM-doc-trastuzumab	>100*	>100*	48.85 ± 4.82*	2.03 ± 0.07*†	0.012 ± 0.005*†	0.004 ± 0.002*†

The IC_50_ values are presented as the mean ± standard deviation of three experiments. *: statistically significant difference towards free drug at (p < 0.05); †: statistically significant difference between cell lines (p < 0.05)

Bold font - remarkable selectivity of PAMAM-doc-trastuzumab conjugate for both breast cancer cell lines

Our results confirm the uniqueness of the PAMAM-drug-trastuzumab conjugates, and reveal a synergistic effect: an increase in the toxic efficiency towards to HER-2-positive cells (SKBR-3) and a decrease in the toxic efficiency towards to HER-2-negative cells (MCF-7). These results present the possibility of significant dose reduction while maintaining the therapeutic effect and selectivity, which can protect from the adverse effects caused by administration of docetaxel or paclitaxel.

This finding is in agreement with a previous study by Rodallec *et al*., which reported the association between cytotoxicity and cellular uptake and the level of HER-2 expression. Immunoliposomes containing docetaxel encapsulated in a stealth liposome and engrafted with trastuzumab showed higher antiproliferative efficacies and efficient drug delivery compared with the standard combination of docetaxel and trastuzumab (22). Furthermore, Kulhari *et al*. confirmed the effectiveness of dendrimer-conjugated monoclonal antibodies and anticancer drugs. Even at very low concentrations (7.8 ng/mL), the trastuzumab-dendrimer-docetaxel conjugate showed significantly higher cytotoxicity against HER-2-positive MDA-MB-453 cells than the dendrimer-docetaxel conjugate, with no significant difference in cytotoxicity observed toward HER-2-negative MDA-MB-231 cells (19). These results demonstrate that trastuzumab can specifically target and successfully deliver docetaxel to HER-2-positive cells.

**Fig. 10:**
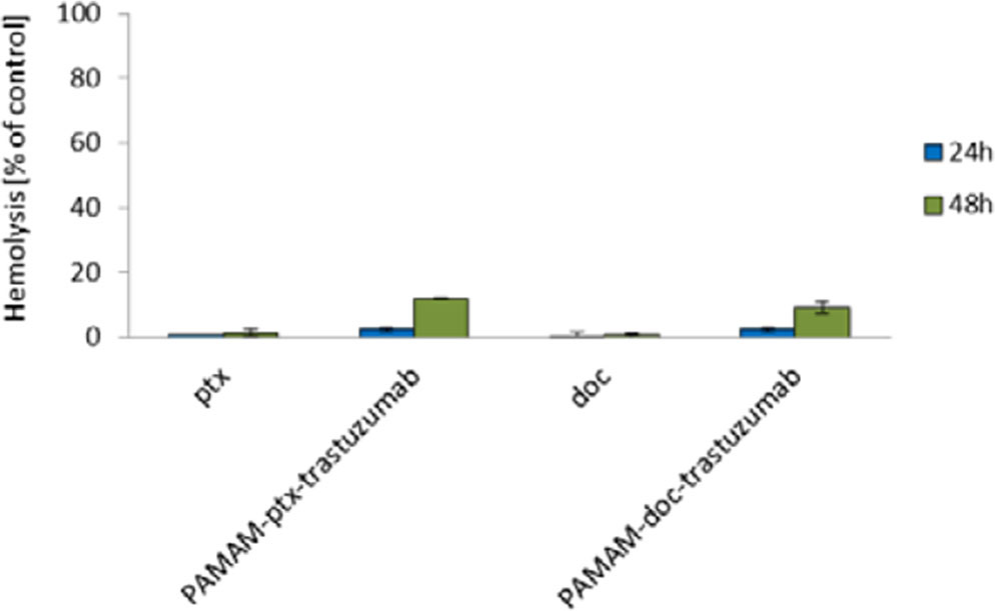
Hemotoxicity of paclitaxel, docetaxel, PAMAM-ptx-trastuzumab and PAMAM-doc-trastuzumab conjugates. The results are presented as the mean ± standard deviation of three experiments

Many clinical trials have shown that intravenous injection is probably the most convenient way to deliver drugs conjugated with dendrimers (23). Unfortunately, very often lysis of red blood cells excludes intravenous delivery of the dendrimer conjugates. PAMAM dendrimers with exposed terminal cationic surface groups possess hemotoxic properties because they are able to disrupt cell membrane of erythrocytes after adhesion to the cell surface by electrostatic attraction and formation of holes in the membrane (24). Modification of dendrimer surface groups is one of the methods used to reduce dendrimer toxicity (15). Therefore, to assess the biocompatibility of the all analysed compounds we have evaluated their hemotoxicityThe ability of the PAMAM-drug-trastuzumab conjugates to cause hemolysis was compared with the hemolytic activity of free drugs (Fig. 10). The paclitaxel and docetaxel are known to possess minor hemolytic properties contrary to amino-terminated PAMAM dendrimer generation 4. Under the applied experimental conditions free drugs caused 1–2% hemolysis after 24 h-incubation. PAMAM-doc-trastuzumab and PAMAM-ptx-trastuzumab conjugates were able to evoke 2.4 and 2.5% hemolysis, respectively. After 48 h of incubation with the above-mentioned conjugates, less than 10% and 12% of hemolysis was observed, respectively. In conclusion, the PAMAM-drug-trastuzumab conjugates possesses higher hemotoxicity than free drugs but it is very likely that this level will be lower in the presence of plasma proteins. Klajnert *et al*. showed that presence of HSA in the same concentration as under physiological conditions significantly reduced the amount of hemolysis caused by PAMAM dendrimers (25). Moreover, the conjugates are not expected to circulate in blood as long as 48 h.

**Fig. 11:**
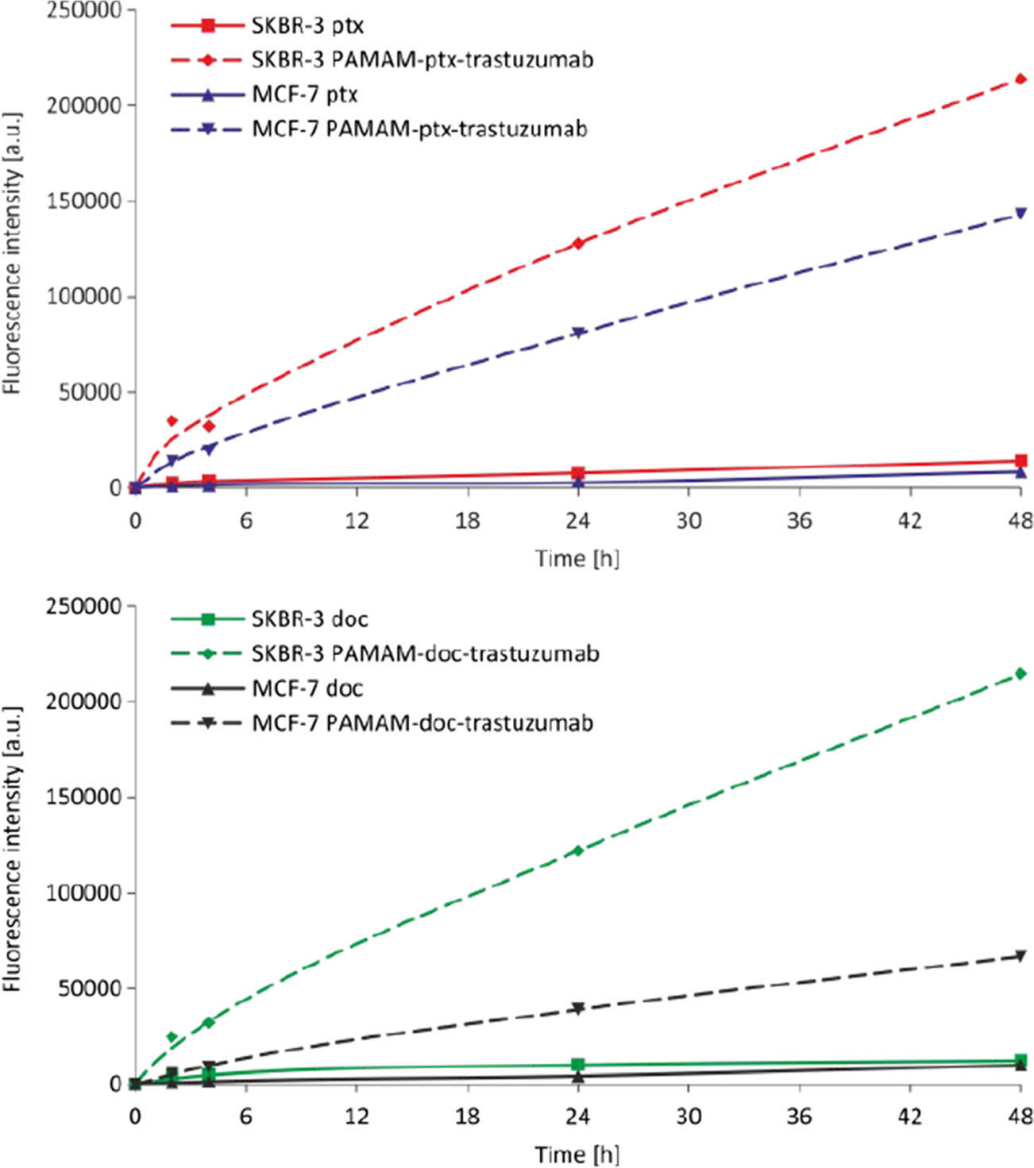
Cellular uptake of free paclitaxel and PAMAM-ptx-trastuzumab conjugate (upper panel) and free docetaxel and PAMAM-doc-trastuzumab conjugate (lower panel) at a concentration of 0.1 μM by MCF-7 and SKBR-3 cells after incubation for 1, 2, 3, 4, 5, 24, and 48 h

In this study, FITC was used to label free drugs and the dendrimer conjugated to anticancer drugs via a pH-dependent linker. The molar ratio of FITC molecules to PAMAM in the conjugate was 1:1. This method of conjugation ensured stability of the conjugates in the extracellular media and high intercellular drug release catalysed by lysosomal enzymes, what resulted in increased antitumour activity. In order to analyse the cellular uptake of free docetaxel, paclitaxel and the PAMAM-drug-trastuzumab conjugates by flow cytometry, cells were incubated with the compounds at concentrations of 0.1 μM for up to 48 h. As expected, the HER-2-positive SKBR-3 cell line accumulated trastuzumab from both conjugates rapidly; but surprisingly, although a large amount of PAMAM-ptx-trastuzumab conjugate was observed in the HER-2-negative MCF-7 cells (Fig. 11). It is likely that the linker-bonded conjugate was less stable in the more acidic environment of the cancer cells, which resulted in earlier paclitaxel release. Importantly, the cellular uptakes of free paclitaxel and docetaxel, even after 48 h of incubation, were significantly lower than the PAMAM-drug-trastuzumab conjugates. This is in good agreement with our results obtained by MTT assay, as well as those of previous studies; for example, Miyano *et al*. suggested that trastuzumab conjugated to the KG6E dendrimer shows HER-2-specific binding, and a consequent high rate of cellular internalisation (9). Other reports have demonstrated the rapid internalisation of trastuzumab-PAMAM (26) and trastuzumab-PLGA (27) nanoparticle conjugates into HER-2-positive breast cancer cells in comparison with free trastuzumab.

**Fig. 12:**
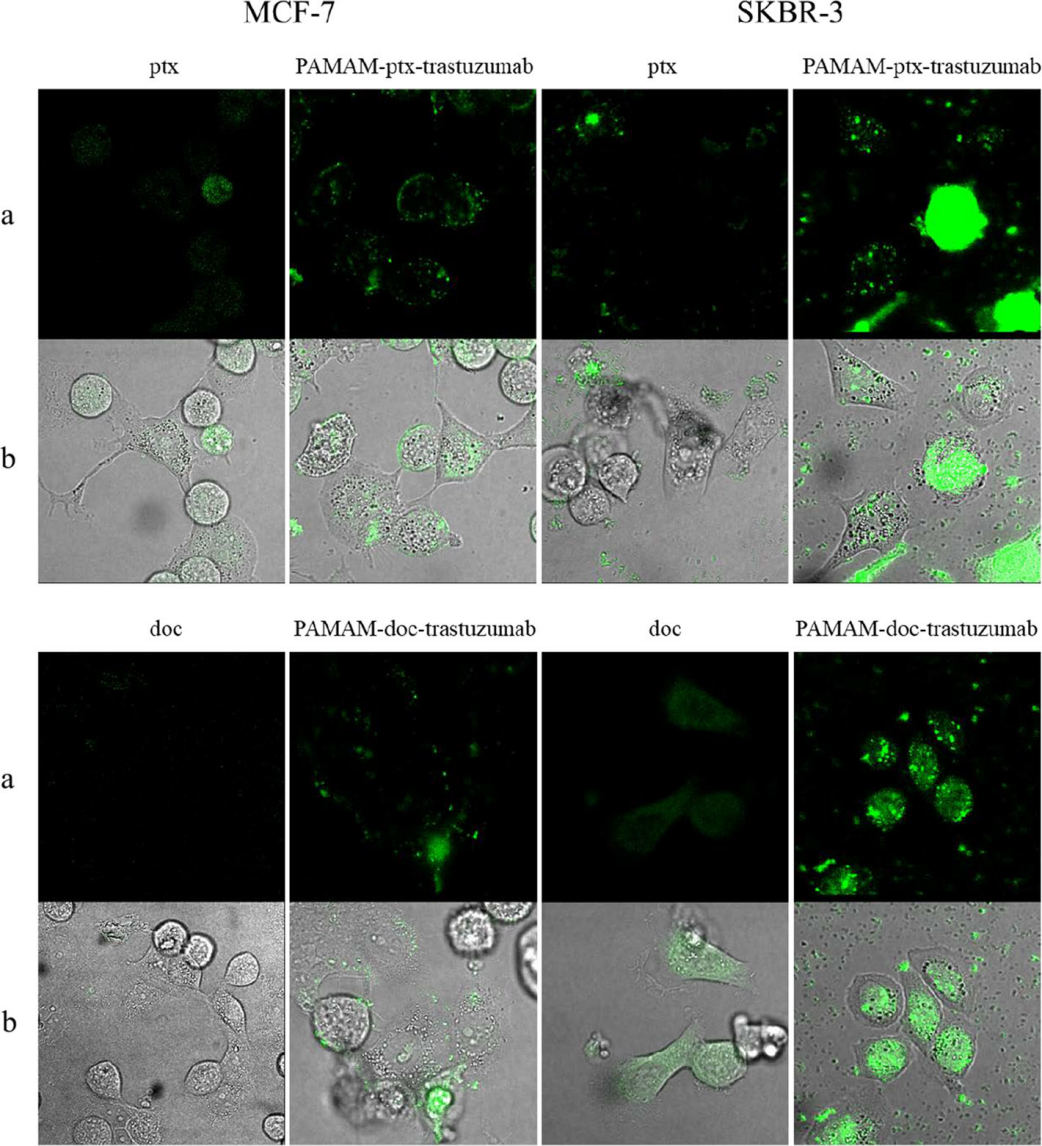
Confocal images of MCF-7 and SKBR-3 cells treated with 0.1 μM free paclitaxel and PAMAM-ptx-trastuzumab conjugate (upper panel) and 0.1 μM free docetaxel and PAMAM-doc-trastuzumab conjugate (lower panel) for 24 h. Paclitaxel, docetaxel and conjugates accumulation imaged using (**a**) the green channel, and (**b**) merged with transmitted light

Confocal microscopy was used to confirm the intracellular localisation of free paclitaxel, docetaxel and the PAMAM-ptx-trastuzumab and PAMAM-doc-trastuzumab conjugates. Incubation of HER-2 positive SKBR-3 and HER-2 negative MCF-7 cells with 0.1 μM of the FITC modified compounds was carried out for 24 h (Fig. 12). Both free drugs were internally localised in both cell lines to some extent; however, paclitaxel was found to be accumulated in the nucleus region in contrast to docetaxel, which was located in the cytosol. Although both conjugates were more concentrated in the nucleus of HER-2-positive compared with negative cells, accumulation of the PAMAM-ptx-trastuzumab conjugate was observed in HER-2-negative cells (mainly in the lysosomes). The key result of fluorescent imaging was the identification of strong selective binding of the PAMAM-doc-trastuzumab conjugate with HER-2-positive SKBR-3 cells only. To the best of our knowledge, this is the first reported example of the visualisation of PAMAM-ptx-trastuzumab and PAMAM-doc-trastuzumab conjugate localisation within HER-2-negative and positive cells.

Our results have a number of similarities with the findings of Ma *et al*. (20). In their study, trastuzumab was covalently linked to a PAMAM dendrimer via a bifunctional PEG linker, and was internalised more efficiently by HER-2-positive BT474 cells than by HER-2-negative MCF-7 cells. Moreover, co-localisation experiments indicated that the trastuzumab-PAMAM conjugate was located in the cytoplasm (20). In other studies, trastuzumab conjugated with the KG6E dendrimer bound selectively to SKBR-3 cells, rather than to MCF-7 cells, although the conjugate was internalised to the lysosomes (9). Rodallec *et al*. confirmed the cellular uptake of docetaxel-trastuzumab stealth immunoliposomes (ANC-1) in different HER-2-positive cell lines, and found that ANC-1 was primarily localised around the cell nuclei. However, ANC-1 showed increased accumulation in SKBR-3 cells compared with the MDA-MB-453 or MDA-MB-231 cell lines (22).

Covalent attachment of a humanised monoclonal antibody trastuzumab to a G5 PAMAM dendrimer containing the drug methotrexate (to form the G5-Fl-HN-MTX conjugate) has been used in the treatment of skin, lung and breast cancer (18). Co-localisation experiments carried out in HER2-expressing MCA207 cell line have indicated that G5-Fl-HN-MTX was localised in the late endosomes and lysosomes within 1 h of exposure, but the most surprising result was the long residence time (48 h) of the conjugate in the lysosomes. This may result in the reduced cytotoxicity which is observed in the case of the G5-Fl-HN-MTX conjugate. Our conjugates are free of such a disadvantage, as it is demonstrated by the results of the MTT assay. Although the steric hindrance caused by covalent conjugation of the antibody to the G5 PAMAM dendrimer may prevent intracellular esterase enzymes from releasing the drug, the conjugate is unable to affect its cytotoxic activities because of the extended retention in the lysosomes. It was for this reason that we decided to use a pH-dependent linker that allows the conjugate to disintegrate in the acidic environment of the cancer cell.

In the literature there are many examples of improved drug delivery as a promising strategy to optimise the effectiveness of anticancer drugs while reducing the toxicity associated with treatment. This study is the first step towards enhancing our knowledge about the design of selective conjugates which can be successfully used for targeted therapy.

## Conclusion

Preclinical studies have demonstrated that HER-2 overexpression occurs in over 20% of breast carcinomas and is associated with resistance to anticancer drugs such as paclitaxel and docetaxel (28). Such studies have also reported the additive effects of synergistic interactions between trastuzumab and taxanes (29). The present study presents the successful synthesis and characterisation of the HER-2-targeted conjugates PAMAM-doc-trastuzumab and PAMAM-ptx-trastuzumab. Analysis of the cytotoxicity, cellular uptake and internalisation of the conjugates indicate that they represent promising carriers for HER-2-expressing tumour-selective delivery. The observed selectivity is achieved not only through the inclusion of trastuzumab, which binds and blocks HER-2, but also through the selection of a pH-sensitive linker that breaks in the tumour environment to allow PAMAM-drug conjugate release. Both conjugates show potential as drug delivery systems enhancing the therapeutic index and reducing the required dosage of anticancer drugs. In our opinion these conjugates might be superior for *in vivo* application due to their increased toxicity for HER-2-positive breast cancer due to specific targeting to tumor cells.

## Electronic supplementary material

ESM 1 (DOCX 168 kb)
