# Supplementary material for: Multicomponent Conjugates of Anticancer Drugs and Monoclonal Antibody with PAMAM Dendrimers to Increase Efficacy of HER-2 Positive Breast Cancer Therapy
Source: Pharm Res. 2019 Sep 3;36(11):154. doi: 10.1007/s11095-019-2683-7 (PMC6722151; doi:10.1007/s11095-019-2683-7)
Supplement: Supplementary file 1 — (DOCX 168 kb) [file 11095_2019_2683_MOESM1_ESM.docx]

**Multicomponent conjugates of anticancer drugs and monoclonal antibody with PAMAM dendrimers to increase efficacy of HER-2 positive breast cancer therapy**

Monika Marcinkowska^1^, Maciej Stanczyk^2^, Anna Janaszewska^1^, Ewelina Sobierajska^1^, Arkadiusz Chworos^3^ and Barbara Klajnert-Maculewicz^1,4,*^

^1^ Department of General Biophysics, Faculty of Biology and Environmental Protection, University of Lodz, Pomorska 141/143, 90-236 Lodz, Poland

^2^ Department of Surgical Oncology, Cancer Center, Copernicus Memorial Hospital, Lodz, Poland

^3^ Centre of Molecular and Macromolecular Studies, Polish Academy of Sciences, Sienkiewicza 112, 90-236 Lodz, Poland

^4^ Leibniz-Institut für Polymerforschung Dresden e.V., Hohe Strasse 6, 01069 Dresden, Germany

***** Correspondence: barbara.klajnert@biol.uni.lodz.pl

***FTIR analysis***

FTIR Spectroscopy is considered a suitable techniques for studying the interactions of drug with polymers. In the our studies we used FTIR spectroscopy to characterize the conjugates based on paclitaxel, docetaxel and PAMAM G4 dendrimers. FTIR spectra were collected using the Nicolet 6700 Series FTIR apparatus equipped with Omnic Program, at resolution of 2cm-1.

The FTIR spectrum of PAMAM-ptx is shown in Figure S1. The main peaks are as follows: N-H and OH stretching vibrations at 3424-3248 cm^-1^, methylene asymmetric and symmetric stretching vibrations at 2916-2869 cm^-1^. The signal connected witch C=O stretching vibration from the ester groups is situated at 1737 cm^-1^. The signal for amide bound is located at 1650 cm^-1^, N-H bending of N-substituted amide at 1439 cm^-1^ and C-C bending at 1355cm^-1^. Ester bond stretching vibrations and C-N stretching vibrations are situated at 1244 cm^-1^ and 1295 cm^-1^ respectively. The aromatic bonds are observed at 1019, 906 and 705 cm^-1^. The presence of new peaks at 951 and 705 cm^-1^ in the FTIR spectra of conjugate show the PAMAM-drug interactions. The peak at 951 cm^-1^ appears at higher frequency than in paclitaxel spectrum and this fact suggests the presence of interactions between the paclitaxel and dendrimers.

**
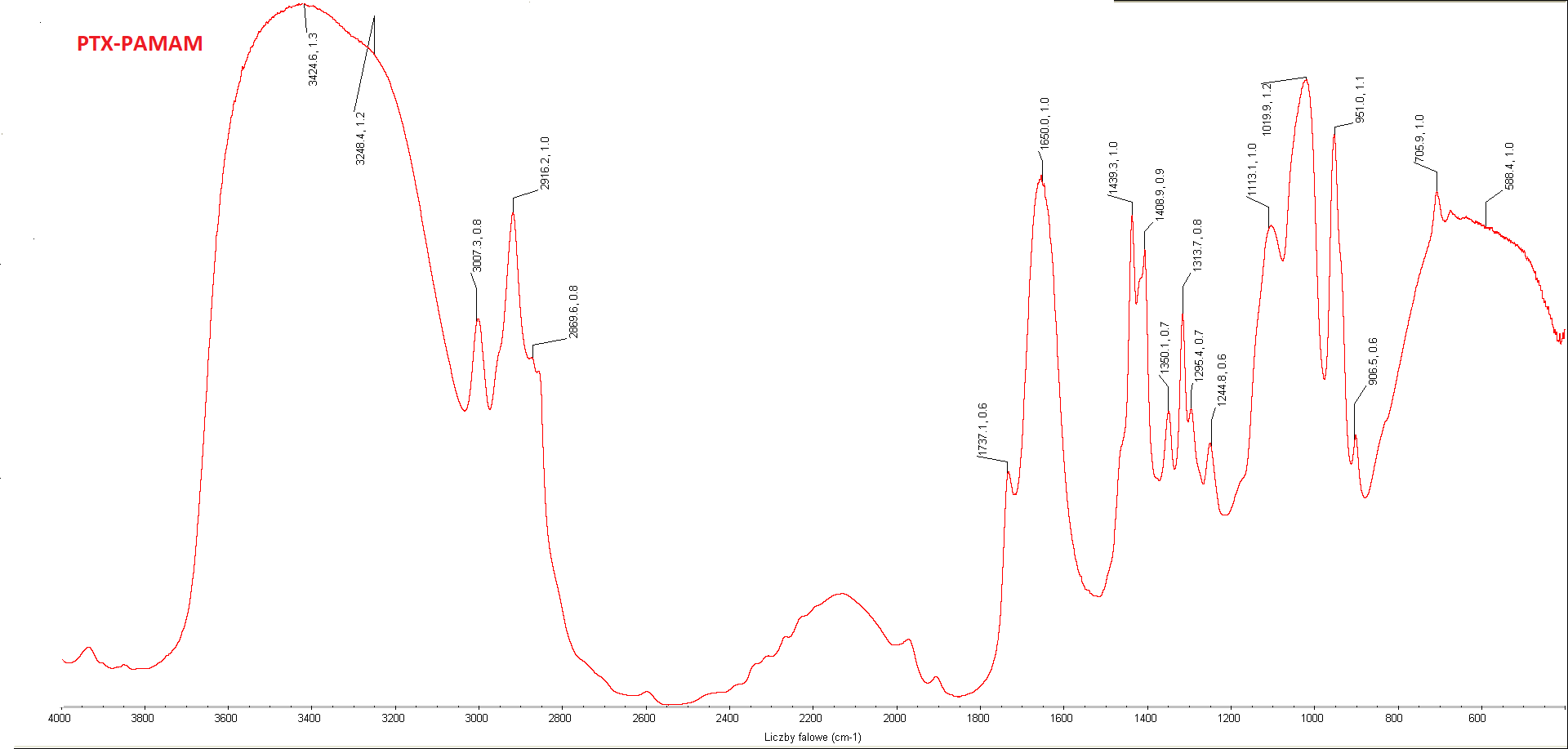
**

**Figure S1.** The FTIR spectrum of PAMAM-ptx.

The FTIR spectrum of PAMAM-doc is shown in Figure S2. The main peaks are as follows: N-H and OH stretching vibrations at 3422cm^-1^, methylene asymmetric and symmetric stretching vibrations around 2920cm^-1^. The amide bound is located at 1647 cm^-1^, N-H bending of N-substituted amide at 1439 cm^-1^ and C-C bending at 1350 cm^-1^. Ester bond stretching vibrations and C-N stretching vibrations are situated at 1248 cm^-1^. A band corresponding to C-O bond is at 1109 cm^-1^. The aromatic bonds are observed at 1019 and 712 cm^-1^. The presence of new signals at 951 and 712 cm^-1^ in the FTIR spectra of conjugate are suggested about the PAMAM-drug interactions.

**
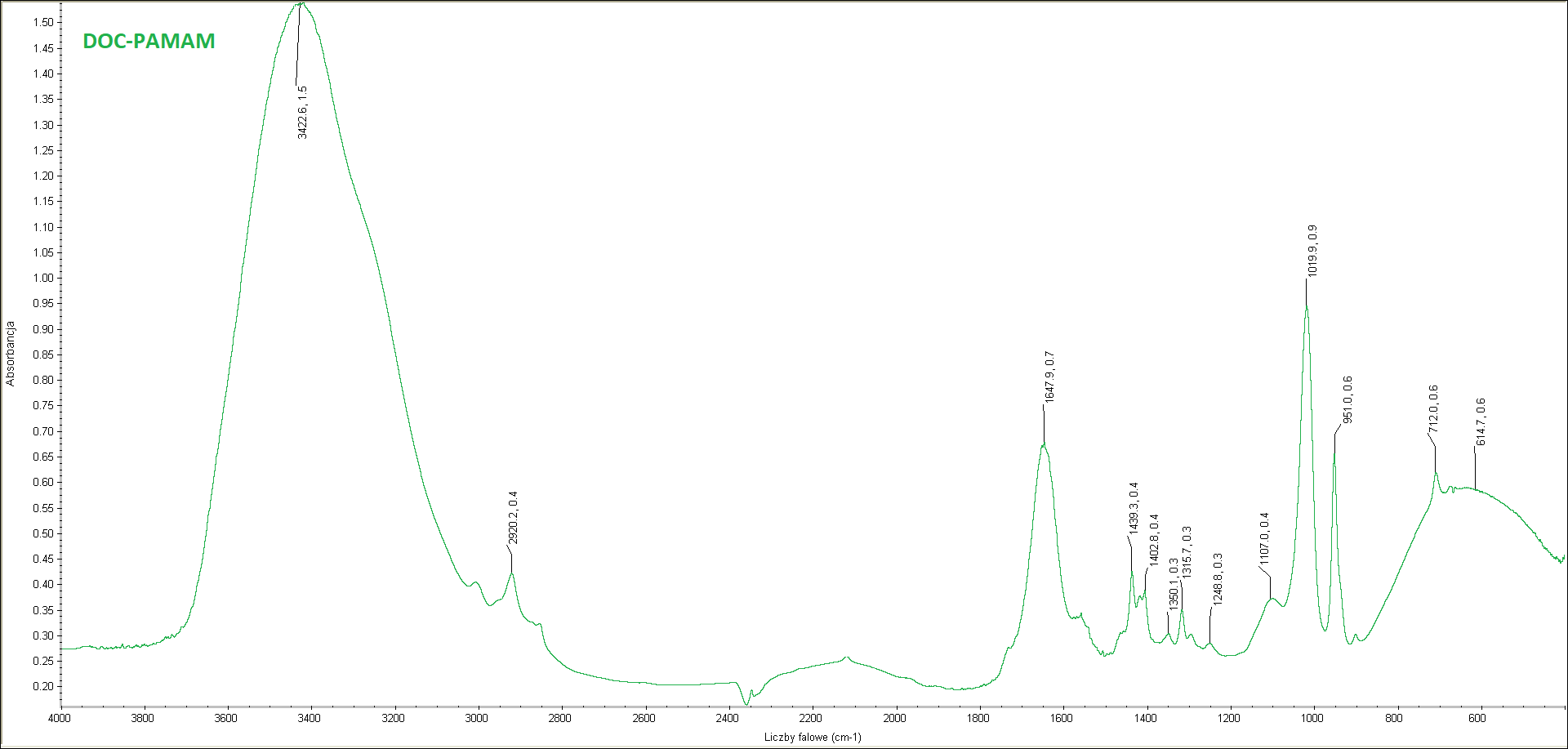
**

**Figure S2.** The FTIR spectrum of PAMAM-doc.

The other peaks detected in the FTIR spectra of PAMAM-ptx and PAMAM-doc conjugates were assigned to the characteristic bonds presented in PAMAM molecules. Assignments of these peaks are summarized in Table S1.

**Table S1.** FTIR analysis for PAMAM-ptx and PAMAM-doc conjugates.

| **Spectral assignments** | **PAMAM-ptx (cm^-1^)** | **PAMAM-doc (cm^-1^)** |
| --- | --- | --- |
| NH stretching vibrations | 3424, 3248 | 3422 |
| CH stretching vibrations | 2916, 2869 | 2920 |
| Amide I mode vibrations | 1737 | 1647 |
| Amide II mode vibrations | 1650 | 1650 |
| CH scissoring vibrations | 1439 | 1439 |
| CH twisting vibrations | 1313 | 1315 |

***DLS analysis***

Additionally, the hydrodynamic diameter (r_h_) of both conjugates was obtained by DLS experiments. Dynamic light scattering (DLS) measurements have been obtained using the Zetasizer Nano ZS (Malvern Instruments, Worcestershire, UK) at T = 37 °C, equipped with a 600 μL quartz batch cuvette (Hellma, Germany). The concentration of the samples was adjusted to 10 µM, to avoid the effect of particle-particle interactions on the diffusion coefficient. Results received previously for trastuzumab were included for comparison purposes (Table S2).

**Table S2.** The experimental results of PAMAM-doc-trastuzumab and PAMAM-ptx-trastuzumab hydrodynamic diameter. The r_h_ values are presented as the mean ± standard deviation of six experiments.

| sample | r_h_ [nm] |
| --- | --- |
| trastuzumab [s1] | 5.20 ± 0.10 |
| trastuzumab [s2] | 5.15 ± 0.08 |
| trastuzumab [s3] | 5.30 ± 0.10 |
| PAMAM-doc-trastuzumab | 14.83 ± 1.60 |
| PAMAM-ptx-trastuzumab | 12.09 ± 0.61 |

| 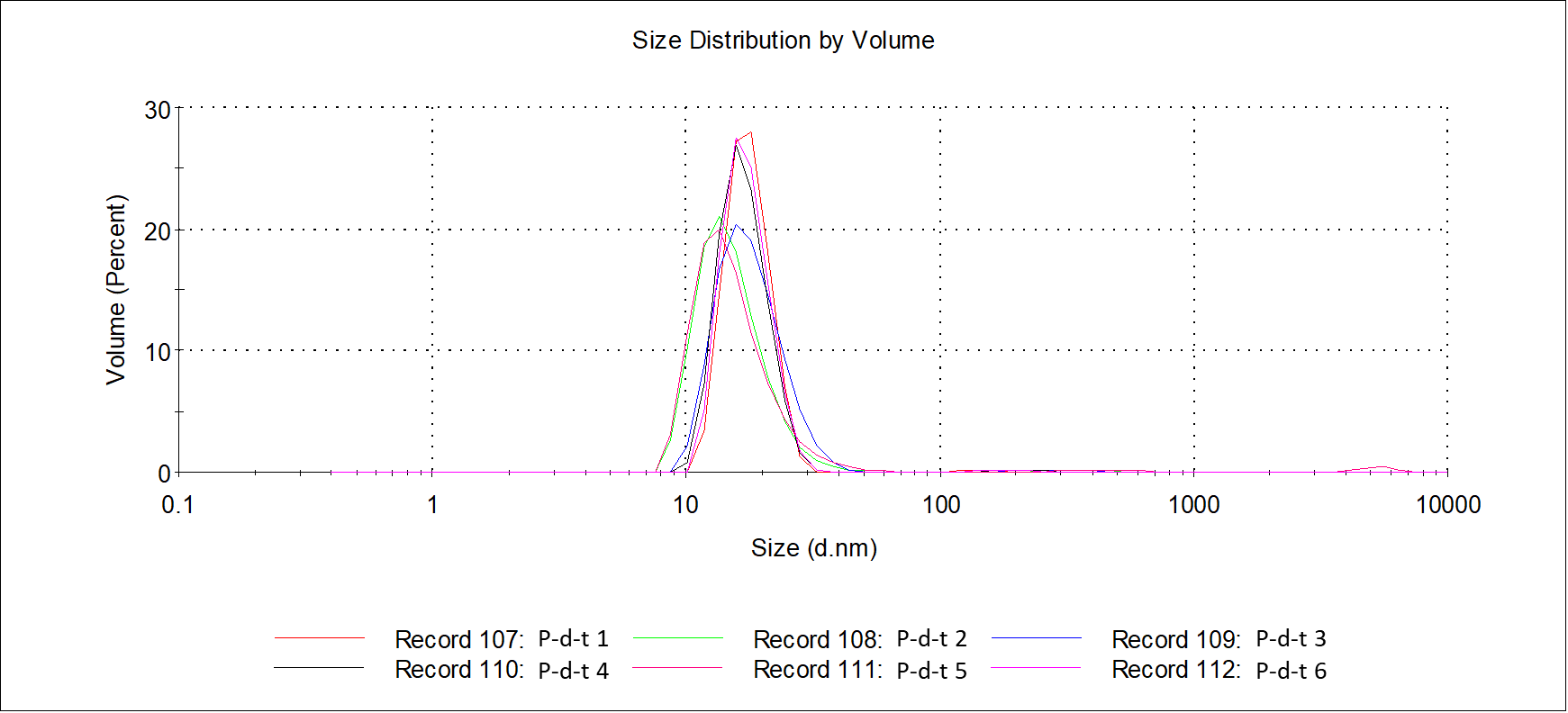 | 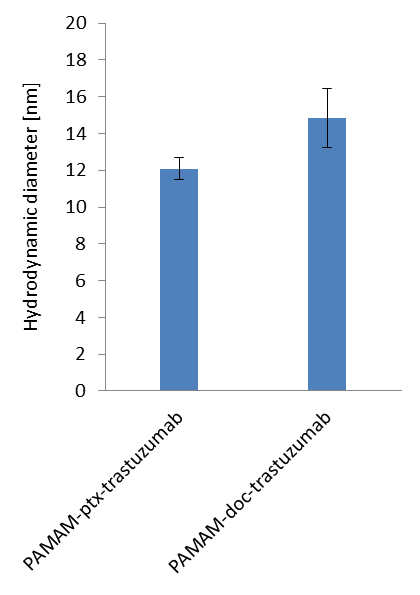 |
| --- | --- |
| 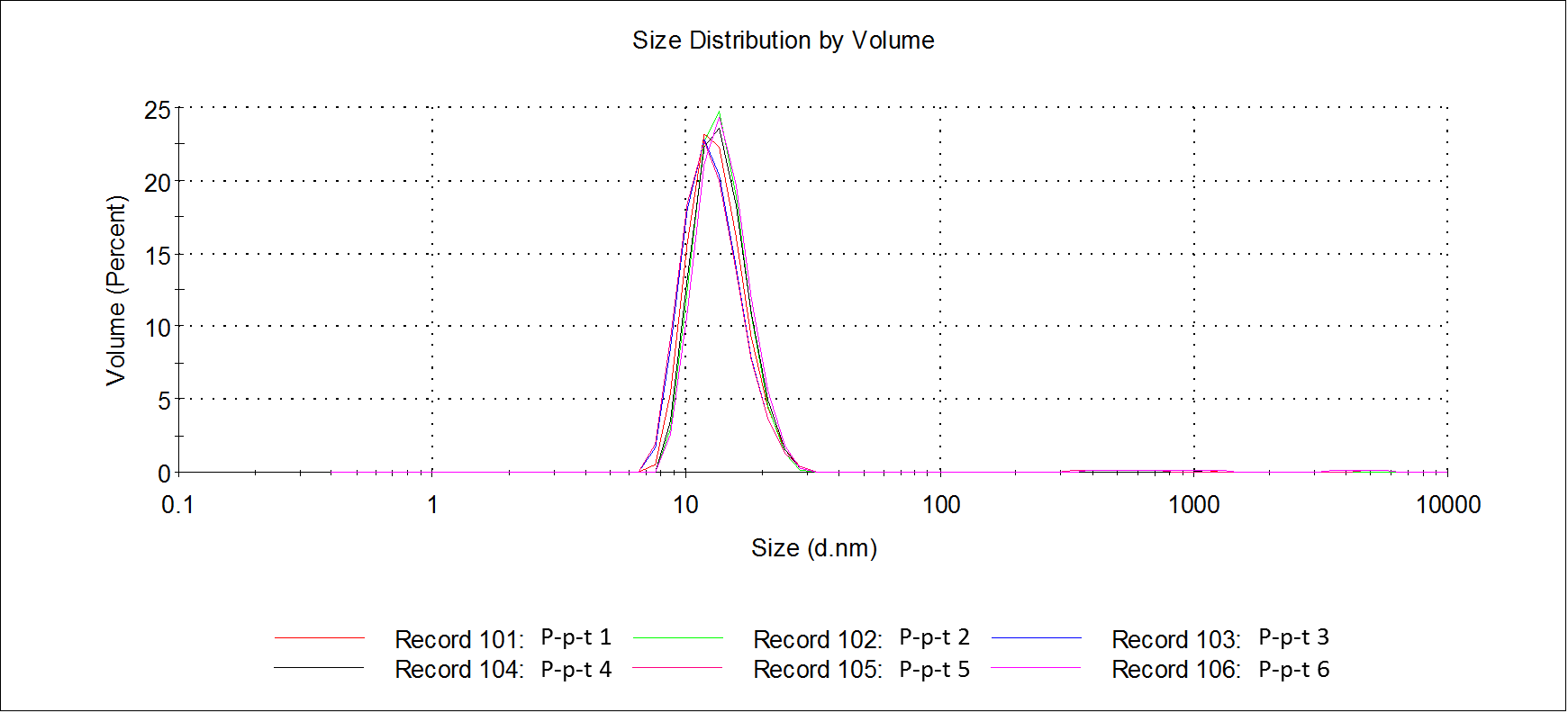 |  |

**Figure S3.** The DLS spectra of PAMAM-doc-trastuzumab (upper) and PAMAM-ptx-trastuzumab (lower).

**Literature**

[s1] Brandt JP, Patapoff TW, Aragon SR. Construction, md simulation, and hydrodynamic validation of an all-atom model of a monoclonal igg antibody. Biophys. J., 99 (2010), 905-913

[s2] Scheer JM, Sandoval W, Elliott JM, Shao L, Luis E, Lewin-Koh SC, Schaefer G, Vandlen R. Reorienting the Fab domains of trastuzumab results in potent HER2 activators. PLoS One. 2012;7(12):e51817

[s3] Espinosa-de la Garza CE, Miranda-Hernández MP, Acosta-Flores L, Pérez NO, Flores-Ortiz LF, Medina-Rivero E. Analysis of therapeutic proteins and peptides using multiangle light scattering coupled to ultra high performance liquid chromatography. J Sep Sci. 2015 May;38(9):1537-43.
